# Clinical outcome of reverse total shoulder arthroplasty (comprehensive system) after failed rotator cuff repair with a medium-term follow-up: comparison with reverse total shoulder arthroplasty for massive rotator cuff tear without osteoarthritis

**DOI:** 10.1016/j.jseint.2025.06.012

**Published:** 2025-07-05

**Authors:** Ji Un Kim, Ji Young Yoon, Young Dae Jeon, Hyung Ki Cho, Hyeon Jang Jeong, Joo Han Oh

**Affiliations:** a Department of Orthopaedic Surgery, Kangwon National University College of Medicine, Kangwon National University Hospital, Chuncheon, Gangwon-do, Republic of Korea; b Department of Orthopaedic Surgery, National Police Hospital, Seoul, Republic of Korea; c Department of Orthopaedic Surgery, Ulsan University College of Medicine, Ulsan University Hospital, Ulsan, Republic of Korea; d Department of Orthopaedic Surgery, Seoul National University College of Medicine, Seoul National University Bundang Hospital, Seongnam, Gyeonggi-do, Republic of Korea

**Keywords:** Massive rotator cuff tear, Reverse total shoulder arthroplasty, Rotator cuff repair, Cuff tear arthropathy, Shoulder, Osteoarthritis, Clinical outcome, Shoulder function

## Abstract

**Background:**

We compared the clinical outcomes of primary reverse total shoulder arthroplasty (rTSA) in patients with massive rotator cuff tears (mRCTs) without osteoarthritis (OA), secondary rTSA in patients with failed rotator cuff repair (RCR), and primary rTSA in patients with cuff tear arthropathy (CTA) as a control group.

**Methods:**

Among 364 patients who underwent rTSA between March 2014 and August 2019, 153 were included. All patients underwent surgery with a single implant type and were followed for a minimum of 4 years. Patients were categorized into three groups: primary rTSA for mRCT without OA (mRCT group, n = 24), primary rTSA for CTA (CTA group, n = 104), and rTSA for failed rotator cuff repair group (fRCR; fRCR group, n = 25). The mean age was 71.5 ± 6.3 (range, 53-83) years, with a mean follow-up of 54.7 ± 12.9 (range, 48-98) months. Functional outcomes were assessed using the active range of motion, the visual analog scale for pain, the simple shoulder test, the American Shoulder and Elbow Surgeons score, the Quick Disabilities of the Arm, Shoulder, and Hand (Q-DASH) score, and the Constant score at the final follow-up.

**Results:**

All functional outcomes significantly improved postoperatively in each group (*P* < .05). However, the fRCR group presented worse outcomes compared to the other groups, including visual analog scale for pain (2.1 ± 0.5), forward flexion (126° ± 4°), external rotation (42 ± 4°), American Shoulder and Elbow Surgeons score (76 ± 5), and Constant score (55 ± 3) (*P* < .05). Postoperative complications and radiologic outcomes were not significantly different between the groups (*P* = .890).

**Conclusion:**

Considering the worse clinical outcomes of secondary rTSA after failed RCR compared to primary rTSA for mRCT without OA and/or CTA, careful selection of appropriate candidates for RCR or primary rTSA as a treatment option for mRCT without OA is essential, according to their healing potential.

The optimal treatment strategy for massive rotator cuff tears (mRCTs) without osteoarthritis (OA) remains debatable.^3^^,^^9^ Although rotator cuff repair (RCR) is considered the treatment of choice, reverse total shoulder arthroplasty (rTSA) has emerged as an alternative treatment option, especially for patients with massive irreparable rotator cuff tears.^9^ Standardized treatment guidelines for failed RCR are lacking.^3^^,^^4^^,^^7^^,^^9^ A previous study indicated a healing failure rate of 39.8% for arthroscopic repair of mRCT.^7^ While short-term functional outcomes after failed RCR are often favorable, long-term outcomes deteriorate over time, with a more rapid progression of arthritic changes compared to that in patients with successful healing.^15^ Furthermore, several studies have reported that muscle atrophy and fatty degeneration in the rotator cuff are typically irreversible, even in cases of successful healing.^11^^,^^20^

For patients with mRCT without OA, superior capsular reconstruction, tendon transfer, or patch grafting may be viable options.^1^^,^^8^^,^^21^^,^^24^^,^^25^^,^^32^ However, these methods exhibit healing failure rates similar to those of RCR, and many debates remain regarding the proper treatment of mRCT with these options.^8^^,^^21^^,^^24^^,^^25^^,^^32^

To address the challenge of healing failure after surgical repair of mRCT, rTSA has been adopted as an alternative. Primary rTSA for mRCT without OA and/or cuff tear arthropathy (CTA) has shown an excellent 10-year survival rate and favorable functional outcomes.^33^ However, rTSA carries unique complications not associated with RCR, including scapular notching, instability, infection, aseptic loosening of the implants, stress fracture of the acromion or scapular spine, and neurologic complications.^40^

Given the lack of clear guidelines for RCR versus rTSA in patients with mRCT without OA, studies comparing primary rTSA for mRCT without OA and secondary rTSA following failed RCR have been rarely conducted.^2^^,^^4^^,^^10^^,^^30^^,^^37^ Therefore, the purpose of this study was to evaluate whether primary rTSA could be an appropriate treatment strategy for patients with mRCT without OA. We compared the clinical outcomes of secondary rTSA following failed RCR and primary rTSA for CTA as a control group. We hypothesized that secondary rTSA after failed RCR would result in worse outcomes than primary rTSA for mRCT without OA and that the outcomes of primary rTSA for mRCT without OA would be similar to those of primary rTSA for CTA.

## Materials and methods

**Figure 1:**
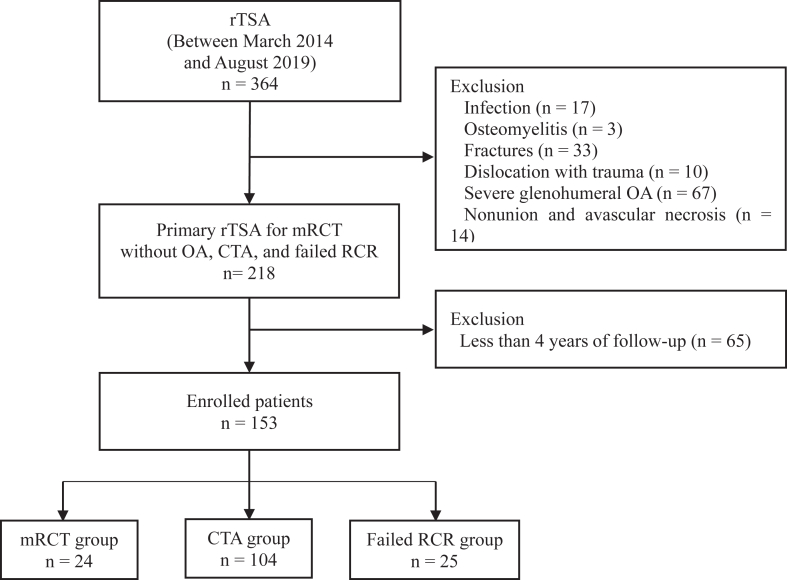
Flowchart depicting the selection process and eligibility of participants included in the current study. *rTSA*, reverse total shoulder arthroplasty; *OA*, osteoarthritis; *mRCT*, massive rotator cuff tear; *CTA*, cuff tear arthropathy; *RCR*, rotator cuff repair.

We retrospectively reviewed 364 patients who underwent rTSA between March 2014 and August 2019, performed by the senior author of this study (J.H.O.), with the approval from the institutional review board of the senior author's affiliation (R-2501-946-103). To minimize bias related to implant morphology, only patients who received a single implant type (Comprehensive System, Zimmer-Biomet, Warsaw, IN, USA) were included (n = 159). Patients with a history of infection (n = 17), osteomyelitis (n = 3), dislocation with trauma (n = 10), fractures (n = 33), and nonunion and/or avascular necrosis (n = 14) on the ipsilateral shoulder, severe OA with small- to medium-sized rotator cuff tear (n = 67), tumor and/or rheumatoid arthritis (n = 2), or those with <48 months of follow-up (n = 65) were excluded (Fig. 1). In this study, a total of 153 patients were enrolled.

We categorized the patients into three groups: primary rTSA for mRCT (mRCT group, n = 24), primary rTSA for CTA (CTA group, n = 104), and secondary rTSA after failed rotator cuff repair group (fRCR; fRCR group, n = 25). mRCT was defined as a rotator cuff tear >5 cm or a complete tear of more than two rotator cuff tendons without OA, verified by preoperative magnetic resonance imaging (MRI).^12^ CTA was defined as Hamada grades 4 or 5, indicating degenerative arthritis with humeral head elevation due to torn rotator cuff tendons.^13^ Failed RCR was identified based on MRI findings following primary RCR.^21^ If the structural integrity of the primary repair site was not maintained in the postoperative MRI (Sugaya type IV or V), the RCR was classified as failed RCR.^35^

Demographic data, clinical symptoms, and functional parameters were collected to compare clinical outcomes across the three groups. Preoperative evaluations were performed the day before surgery, and postoperative evaluations were conducted annually in an outpatient clinic. The results of the most recent follow-up were used for analysis.

Demographic data included sex, age at the time of operation, body mass index, dominant hand, bone mineral density, and levels of sports and work activity. To evaluate clinical symptom improvement, the interval between symptom onset and surgery, the presence of pseudoparalysis, and the external rotation (ER) lag sign were investigated.

Functional status was evaluated using the active range of motion measurements, including forward flexion (FF), ER of the arm at the side, and internal rotation (IR) of the arm at the back. Additional measures included the visual analog scale for pain (pVAS), the simple shoulder test (SST), the American Shoulder and Elbow Surgeons (ASES) Assessment Form, and the Constant score. FF and ER were evaluated using a goniometer, while IR was measured based on the highest vertebral level the thumb could reach in a seated position. For statistical analysis, IR was converted to a continuous numerical scale: T1–12 (1–12), L1–5 (13–17), and levels below the sacrum.^18^ Postoperative complications, such as notching, stress fractures, and dislocations, as well as radiological outcomes, were assessed and compared among the groups.

All surgical procedures were performed under general anesthesia without differences among the three groups. The glenohumeral joint was exposed through a deltopectoral approach with the patient in the beach chair position. The subscapularis tenotomy was performed longitudinally at 1 cm medial to the bicipital groove. The humeral neck was cut according to the patient's native retroversion, which was preoperatively measured using a computed tomography (CT) scan, including the proximal and distal portions of the humerus.^29^ Glenoid preparation involved placing a guide pin at the inferior rim of the baseplate onto the inferior rim of the bony glenoid, with a 10-15° inferior tilt.^27^

Glenoid reaming was performed along the guide pin to create a neutral version. All patients received a baseplate with a 25-mm diameter and a 36-mm glenosphere featuring a 3.5-mm inferior offset. The thickness of the humeral tray and insert was determined according to implant stability and soft tissue tension, which were evaluated by trial reduction.^23^ If feasible, the subscapularis was reattached transosseously using three strands of No. 2 Ethibond sutures (Ethicon, Raritan, NJ, USA), followed by tenodesis of the long head of the biceps tendon along with the repaired adjacent subscapularis.

All patients were immobilized with an abduction brace for 4 weeks from the day of the surgery. Passive FF exercises using a continuous passive motion machine were encouraged during immobilization. The active-assisted stretching exercise was started 4 weeks postoperatively after weaning of the brace. Usually, rotator cuff and deltoid muscle strengthening exercises are introduced 3 months postoperatively, and all sports activities are allowed 6 months postoperatively. Patients were advised to avoid heavy lifting, strenuous activities involving the operative arm, and contact sports. This information is included to provide postoperative activity restrictions and enhance understanding of the recommended rehabilitation protocol.

The statistical analysis in this study involved comparisons among the three treatment groups. The Shapiro–Wilk normality test and Levene's homogeneity test were conducted first. If both tests indicated normality and homogeneity, an analysis of variance was performed. In cases where at least one of the tests was not met, the Kruskal–Wallis test was performed instead. For count data, Pearson's chi-squared test or Fisher's exact test was performed based on the frequency distribution. Multiple comparisons were performed to identify statistically significant differences between groups. Finally, the Benjamini–Hochberg test was applied as a post hoc analysis to ascertain which specific comparisons among the groups yielded significant results. Paired observations before and after surgery were compared using a paired t-test. Statistical significance was set at *P* < .05, with adjusted *P* values in the post hoc tests for clarity. To evaluate the clinical relevance of statistical differences, we set the minimal clinical important differences (MCID) of pVAS, SST, ASES, and Constant score as 1.4, 1.4, 10.3, and 9.3, respectively.^31^^,^^39^ All statistical analyses were performed using SPSS software (version 21.0; IBM Corp., Armonk, NY, USA).

## Results

The mean age at the time of surgery was 71.5 ± 6.3 (range, 53-86) years, while the mean follow-up period was 54.7 ± 12.9 (range, 48-98) months. The demographic data (Table I) and preoperative functional status (Table II) were not significantly different between the groups (all *P* > .05).

**Table I:** Demographic characteristics of the study population.

	mRCT (n = 24)	CTA (n = 104)	Failed RCR (n = 25)	*P* value
Sex, male: female	6:18	19:85	6:19	.583
Age, yr	70.2 ± 5.8	71.5 ± 6.2	71.8 ± 7.3	.380
BMI, kg/m^2^	25.8 ± 3.1	25.5 ± 3.5	24.7 ± 3.4	.463
Involved side, dominant hand: nondominant hand	21:3	75:29	22:3	.455
BMD, T-score	−1.8 ± 1.2	−2.0 ± 0.9	−2.3 ± 1.6	.422
Pseudoparalysis, n (%)	6 (25%)	46 (44%)	7 (28%)	.205
ER lag sign, n (%)	6 (25%)	35 (34%)	5 (20%)	.479
Onset, yr	5.6 ± 6.7	6.2 ± 7.3	6.7 ± 7.9	.397
Level of sports, low: medium: high	23:1:0	102:2:0	25:0:0	.519
Level of work, low: medium: high	21:3:0	84:14:9	18:7:0	.197

*mRCT*, massive rotator cuff tear; *CTA*, cuff tear arthropathy; *RCR*, rotator cuff repair; *BMI*, body mass index; *BMD*, bone mineral density; *ER*, external rotation.

Data are presented as means ± standard deviations, ratios, or numbers (percentages).

**Table II:** Preoperative and postoperative comparison of clinical outcomes in each group.

	mRCT			CTA			Failed RCR		
	Preoperative	Postoperative	*P* value	Preoperative	Postoperative	*P* value	Preoperative	Postoperative	*P* value
pVAS	5.5 ± 0.5	0.8 ± 0.3	<.001∗	6.1 ± 0.2	1.1 ± 0.2	<.001∗	6.7 ± 0.4	2.1 ± 0.5	<.001∗
FF, °	114 ± 12	142 ± 4	.019∗	127 ± 4	149 ± 1	<.001∗	106 ± 9	126 ± 4	.018∗
ER, °	39 ± 5	51 ± 4	.061	42 ± 2	60 ± 2	<.001∗	36 ± 4	42 ± 4	.253
IR, VL†	10 ± 1	10 ± 1	.257	11 ± 0	9 ± 0	<.001∗	13 ± 1	10 ± 1	.010∗
ASES	47 ± 4	90 ± 3	<.001∗	42 ± 2	87 ± 2	<.001∗	36 ± 4	76 ± 5	<.001∗
SST	2 ± 0	9 ± 1	<.001∗	2 ± 0	9 ± 0	<.001∗	2 ± 1	7 ± 1	<.001∗
Constant	44 ± 3	64 ± 3	<.001∗	44 ± 1	64 ± 1	<.001∗	38 ± 3	55 ± 3	<.001∗

*mRCT*, massive rotator cuff tear; *CTA*, cuff tear arthropathy; *RCR*, rotator cuff repair; *pVAS*, visual analog scale for pain; *FF*, forward flexion; *ER*, external rotation; *IR*, internal rotation; *VL*, vertebral level; *ASES*, American Shoulder and Elbow Surgeons score; *SST*, simple shoulder test.

Data are presented as means ± standard deviations.

∗ Statistically significant.

† Internal rotation was converted to a continuous number; T1–12 (1–12), L1–5 (13–17), and below sacrum (18).

At the final follow-up, functional status had improved after surgery in all three groups (all *P* < .05, Table II). As functional outcomes at final follow-up showed differences between the groups except for IR and SST (all *P* < .05, Table III), we conducted post hoc analyses. While functional outcomes at the final follow-up were not different between the mRCT and CTA groups (all *P* > .05), the fRCR group showed worse outcomes in pVAS (fRCR vs. mRCT vs. CTA 2.1 ± 0.5 vs. 0.8 ± 0.3 vs. 1.1 ± 0.2, *P* value), FF (126° ± 4° vs. 142° ± 4° vs. 149° ± 1°, *P* value), ER (42° ± 4° vs. 51° ± 4° vs. 60° ± 2°, *P* value), ASES score (76 ± 5 vs. 90 ± 3 vs. 87 ± 2, *P* value), and Constant score (55 ± 3 vs. 64 ± 3 vs. 64 ± 1, *P* value) compared to those in the mRCT and CTA groups (Table III). In post hoc analyses, the ASES score in the failed RCR group presented clinically meaningful differences exceeding the MCID threshold (10.3).^31^ However, the pVAS (1.4) and Constant score (9.3) did not surpass their respective MCID values.^39^

**Table III:** Comparison of postoperative clinical outcomes between study groups.

	mRCT	CTA	Failed RCR	*P* value∗
pVAS	0.8 ± 0.3	1.1 ± 0.2	2.1 ± 0.5	.046†
*P* value‡	.575	-	.056	
*P* value§	.039†	.056	-	
FF, °	142 ± 4	149 ± 1	126 ± 4	<.001†
*P* value‡	.098	-	<.001†	
*P* value§	.009†	<.001†	-	
ER, °	51 ± 4	60 ± 2	42 ± 4	<.001†
*P* value‡	.06	-	<.001†	
*P* value§	.093	<.001†	-	
IR‖	10 ± 1	9 ± 0	10 ± 1	.061
ASES	90 ± 3	87 ± 2	76 ± 5	.012†
*P* value‡	.401	-	.036†	
*P* value§	.017†	.036†	-	
SST	9 ± 1	9 ± 0	7 ± 1	.070
Constant	64 ± 3	64 ± 1	55 ± 3	<.001†
*P* value‡	.831	-	.002†	
*P* value§	.022†	.002†	-	

*mRCT*, massive rotator cuff tear; *CTA*, cuff tear arthropathy; *RCR*, rotator cuff repair; *pVAS*, visual analog scale for pain; *FF*, Forward flexion; *ER*, external rotation; *IR*, internal rotation; *ASES*, American Shoulder and Elbow Surgeons score; *SST*, simple shoulder test.

Data are presented as means ± standard deviation. Post hoc analysis was performed only if the comparison between the three groups presented statistically significant differences.

∗ Comparison between three groups.

† Statistically significant.

‡ Post hoc comparison with CTA.

§ Post hoc comparison with failed RCR.

‖ Internal rotation was converted to a continuous number; T1–12 (1–12), L1–5 (13–17), and sacrum (18).

Postoperative complications occurred in seven shoulders from seven patients, with no significant differences in postoperative complication rates between the three groups (*P* = .890). The mRCT group had 1 case of scapular notching (4.1%) and 1 case of intraoperative periprosthetic humeral fracture (4.1%), both of which healed spontaneously during the immobilization period. The CTA group had two cases of scapular notching (1.9%). The fRCR group included two cases of scapular notching (8.0%) and 1 case of humeral tray breakage (4.0%). There were two revision cases of rTSA due to scapular notching in the CTA group and humeral tray breakage in the fRCR group. Notably, complications, such as instability and stress fractures, which have been reported in previous studies,^37^^,^^40^ were not observed in any of the groups.

## Discussion

Although the functional status of all three groups improved following rTSA, significant differences in postoperative functional outcomes were observed among them. In the primary rTSA group, functional outcomes were comparable between the mRCT and CTA groups. However, secondary rTSA performed as salvage procedure for failed RCR resulted in worse functional outcomes than primary rTSA for either mRCT or CTA.

While RCR often yields better functional outcomes and a lower complication rate compared to rTSA for treatment of mRCT without OA,^3^^,^^22^ its long-term outcomes may be limited by the risk of healing failure. To address this challenge, various joint-preserving procedures, such as superior capsular reconstruction,^25^^,^^32^ patch augmentation,^6^^,^^8^ balloon spacer,^34^ tendon transfer,^5^^,^^16^^,^^26^ and biceps rerouting^17^ have been investigated and shown favorable short-term outcomes. However, their effectiveness is highly dependent on patient selection and procedure-related complications.^16^ If revision to rTSA after failure of these procedures could yield outcomes equivalent to those of primary rTSA, these joint-preserving procedures might remain a viable initial treatment option. However, as demonstrated in the current study, secondary rTSA following failed RCR results in less favorable outcomes compared to primary rTSA. Therefore, careful patient selection based on accurate preoperative assessment is essential when considering RCR as the treatment option for patients with high risk of healing failure.

Considering the less favorable outcomes associated with secondary rTSA following failed RCR, primary rTSA may be an appropriate initial treatment option even in patients with mRCT without OA. To evaluate its effectiveness in these indications, we selected primary rTSA for CTA as a control group for comparison with mRCT without OA and failed RCR. This selection was based on the rationale that rTSA was originally developed to overcome the limitations of RCR, particularly in the management of CTA, where it has demonstrated excellent long-term outcomes, with revision rates <10%.^3^^,^^33^ CTA is widely recognized as the gold standard indication for rTSA, consistently yielding favorable outcomes.^9^^,^^18^^,^^28^^,^^36^^,^^38^

Previous studies have reported comparable clinical outcomes between primary rTSA performed for mRCT and for CTA.^36^ In contrast, secondary rTSA, performed as a salvage procedure after failed RCR, has been associated with higher complication and revision rates, despite its potential to improve functional outcomes.^2^ Furthermore, several studies have reported that functional outcomes of secondary rTSA are inferior to those of primary rTSA, similar to the current study findings.^4^^,^^10^^,^^37^

Several anatomical and technical factors may contribute to the inferior outcomes observed in secondary rTSA following failed RCR. Although arthroscopic RCR is a minimally invasive procedure, it can still result in partial deltoid detachment or atrophy in some cases.^39^ Additionally, postoperative soft tissue scarring may lead to joint stiffness, limiting intraoperative exposure and compromising accurate implant positioning.^18^^,^^40^ These factors, along with reduced range of motion and diminished muscle strength, can hinder effective rehabilitation and ultimately lead to suboptimal functional outcomes.

Moreover, a prior surgical history may increase the risk of postoperative complications. Notably, patients undergoing rTSA after failed RCR reportedly have a significantly higher risk of periprosthetic joint infection compared to those without previous surgical intervention.^14^ While our study did not identify significant differences in overall complication rates among the three groups, the secondary rTSA group for failed RCR demonstrated inferior functional outcomes compared to the primary rTSA groups for both mRCT and CTA.

Therefore, predicting the healing potential of RCR is a critical component of the surgical decision-making process in patients with mRCT without OA. Although the validation of healing prediction tools is beyond the scope of this study, several scoring systems such as the rotator cuff healing index may assist in identifying patients at high risk of healing failure.^19^^,^^23^ Given the inferior outcomes observed in the secondary rTSA group, such tools may aid in guiding surgical decision-making and support the selection of primary rTSA in appropriately selected patients.

Our study has the advantage of directly comparing clinical outcomes between primary rTSA for mRCT and secondary rTSA following failed RCR,^2^^,^^4^^,^^10^^,^^30^^,^^37^ and it is further distinguished by the inclusion of primary rTSA for CTA as a control group, an established indication for primary rTSA. This methodological approach enhances the clinical relevance and generalizability of our findings.

However, several limitations should be acknowledged. First, the retrospective design of this study inherently carries the risk of selection and reporting bias. To mitigate this, we attempted to minimize heterogeneity by including only patients treated with a single implant type and by excluding cases with potential confounding factors. Nevertheless, the relatively small sample size and limited follow-up duration may affect the generalizability and long-term applicability of our findings. For similar reasons, the failed RCR group was inherently heterogeneous. Although preoperative variables and the specific causes of RCR failure may have influenced subsequent rTSA outcomes, these factors were not systematically analyzed. To address this limitation, multicenter studies with larger cohorts and longer follow-up periods are warranted.

Second, while this study focused on comparing rTSA outcomes based on surgical indication, it did not include a direct comparison with RCR or other joint-preserving procedures. Such comparisons were deemed beyond the scope of this investigation and may have introduced additional heterogeneity, thereby compromising the internal validity of the analyses. A prospective, randomized controlled trial would be more appropriate to address this question.

Finally, all procedures were performed using a single implant system with a standardized glenosphere size and offset. While this approach minimized confounding related to implant variability, it also limits the generalizability of the findings. Further research comparing different implant designs and configurations is necessary to validate and extend these results.

## Conclusion

Given the poorer clinical outcomes of secondary rTSA after failed RCR compared to that via primary rTSA for mRCT without OA and/or CTA, it is essential to carefully select appropriate candidates for either RCR or primary rTSA as treatment options for mRCT without OA, based on the healing potential of the rotator cuff tendons. However, to enhance the generalizability of these findings, further studies comparing various rTSA implant designs are warranted to validate the outcomes observed with the Comprehensive System.

## Acknowledgment

The authors express their gratitude to Editage (www.editage.co.kr) for their assistance with English-language editing.

## Disclaimers:

Funding: No funding was disclosed by the authors.

Conflicts of interest: The authors, their immediate families, and any affiliated research foundations have not received any financial compensation or other benefits from any commercial entities related to the subject of this article.
